# The southern Gulf of Mexico: A baseline radiocarbon isoscape of surface sediments and isotopic excursions at depth

**DOI:** 10.1371/journal.pone.0231678

**Published:** 2020-04-15

**Authors:** Samantha H. Bosman, Patrick T. Schwing, Rebekka A. Larson, Natalie E. Wildermann, Gregg R. Brooks, Isabel C. Romero, Joan-Albert Sanchez-Cabeza, Ana Carolina Ruiz-Fernández, Maria Luisa Machain-Castillo, Adolfo Gracia, Elva Escobar-Briones, Steven A. Murawski, David J. Hollander, Jeffrey P. Chanton

**Affiliations:** 1 Department of Earth, Ocean and Atmospheric Science, Florida State University, Tallahassee, Florida, United States of America; 2 College of Marine Science, University of South Florida, Saint Petersburg, Florida, United States of America; 3 Eckerd College, Saint Petersburg, Florida, United States of America; 4 Instituto de Ciencias del Mar y Limnologia, Universidad Nacional Autonoma de Mexico, Ciudad de México, Mexico; Southern Illinois University, UNITED STATES

## Abstract

The southern Gulf of Mexico (sGoM) is home to an extensive oil recovery and development infrastructure. In addition, the basin harbors sites of submarine hydrocarbon seepage and receives terrestrial inputs from bordering rivers. We used stable carbon, nitrogen, and radiocarbon analyses of bulk sediment organic matter to define the current baseline isoscapes of surface sediments in the sGoM and determined which factors might influence them. These baseline surface isoscapes will be useful for accessing future environmental impacts. We also examined the region for influence of hydrocarbon deposition in the sedimentary record that might be associated with hydrocarbon recovery, spillage and seepage, as was found in the northern Gulf of Mexico (nGoM) following the Deepwater Horizon (DWH) oil spill in 2010. In 1979, the sGoM experienced a major oil spill, Ixtoc 1. Surface sediment δ^13^C values ranged from -22.4‰ to -19.9‰, while Δ^14^C values ranged from -337.1‰ to -69.2‰. Sediment δ^15^N values ranged from 2.8‰ to 7.2‰, while the %C on a carbonate-free basis ranged in value of 0.65% to 3.89% and %N ranged in value of 0.09% to 0.49%. Spatial trends for δ^13^C and Δ^14^C were driven by water depth and distance from the coastline, while spatial trends for δ^15^N were driven by location (latitude and longitude). Location and distance from the coastline were significantly correlated with %C and %N. At depth in two of twenty (10%) core profiles, we found negative δ^13^C and Δ^14^C excursions from baseline values in bulk sedimentary organic material, consistent with either oil-residue deposition or terrestrial inputs, but likely the latter. We then used ^210^Pb dating on those two profiles to determine the time in which the excursion-containing horizons were deposited. Despite the large spill in 1979, no evidence of hydrocarbon residue remained in the sediments from this specific time period.

## Introduction

The southern Gulf of Mexico (sGoM) is a diverse ecosystem of lagoons, river catchments, and shallow shelves, and is home to many important economic activities. The coastal areas are densely populated, and tourism and recreational fishing partly support the population [1]. The two most important economic drivers are the fishing industry and the oil industry [2]. Several fisheries have been important, including brown shrimp (*Farfantepenaeus aztecus*), white shrimp (*Litopenaeus setiferus*), pink shrimp (*Farfantepenaeus duorarum*), maya octopus (*Octopus maya*), red grouper (*Epinephelus morio*), snook (*Centropomus spp*.), and the brackish water clams (*Rangia cuneata*, *Polymesoda carolineana*) [3]. Prior to the discovery of oil fields in the late 1970s and 1980s [4], the pink and white shrimp industries contributed largely to the economy but these fisheries have since collapsed [5]. Exploration for oil reached the region in the early 1950’s and oil exploitation in the 1970’s [1]. The sGoM is now home to a robust oil recovery infrastructure, with over 75% of Mexican oil production occurring in the region [6]. Offshore oil exploitation began in 1976 and by 2016, the area had 256 platforms, with a crude oil production of 1,701,000 barrels per day [1].

As a result of these expanding oil recovery efforts, the area has experienced some oil spills over the years [7]. The most notable spill was the 1979 Ixtoc 1 oil spill that released an estimated 475,000 metric tons of oil [8]. This spill was the largest accidental spill at the time and now ranks second, following the 2010 Deepwater Horizon (DWH) spill [9, 10]. During the Ixtoc 1 spill, oil-residue reached the Texas shoreline [11], after reaching the coastal areas of Campeche, Tabasco and Veracruz in the coastal zones of Mexico. It was estimated that 25–33% of the oil sank to the seafloor [12, 13].

Measurements of bulk isotopic composition have been successfully used to determine quantities of oil-residue in the environment. This approach is referred to as “inverse tracing” because rather than adding a “hot” or radioactive label, the oil spill adds a radiocarbon depleted or ^14^C-dead signal [14–16]. When oil is released to the environment, it undergoes biodegradation, weathering and oxygenation processes which alter the material’s chemical structure, but not its isotopic composition. The isotopic approach is unique because it does not rely on the identification of specific petroleum structures [17, 18]. For example, White et al. [16] observed that the difference between petrocarbon determined by ^14^C mass balance and the sum of petroleum hydrocarbons in sediment that were quantified using flame ionization gas chromatography (GC) increased because of changes in the petroleum fraction into a non GC amenable residue. White et al. [15] used radiocarbon to detect and quantify petroleum residues in the sediments of Wild Harbor, West Falmouth, MA, that have remained there for more than 30 years following a spill. Reddy et al. [14] used ^14^C to discriminate between carbon sources to polycyclic aromatic hydrocarbon and black carbon and found that they were mostly derived from fossil carbon. Radiocarbon is a more powerful tracer than ^13^C isotopic composition because of the greater difference in the two scales [19]. δ^13^C values vary from approximately -20‰ for open ocean production versus oil which is around -27‰, while Δ^14^C values vary from -40‰ to -1000‰ for these same sources [19].

The seafloor may receive inputs of petrocarbon or oil-residue following an oil spill. A large marine snow event was documented during the DWH spill, which resulted in a rapid sedimentation pulse [20, 21]. Marine snow consists of materials such as phytoplankton, bacteria or detritus, that collide and stick together [20]. When the material binds with buoyant or non-buoyant weathered oil, it may sink to the seafloor. This process was later termed MOSSFA (Marine Oil Snow Sedimentation and Flocculent Accumulation) [22, 23]. A similar sedimentation event was described for the Ixtoc 1 oil spill (e.g. [12, 24]). Patton et al. [24] described pancake shaped mousse of oil at the surface, and Jernelöv and Lindén [12] described a 1–4 cm thick layer of emulsified oil at the surface that gradually increased in density as it accumulated particles and weathered until it sank to the sea floor. Vonk et al. [25] performed a meta-analysis on a number of major oil spills and concluded that an oil-residue sedimentation event likely occurred during the Ixtoc 1 oil spill. Jernelöv and Lindén [12] estimated that 50% of the Ixtoc 1 oil had evaporated into the atmosphere, 12% was biologically and chemically degraded, 5% was mechanically removed, and 25% sank to the seafloor.

Baseline data on sediment isotopic composition provides vital information that can be used to determine the impacts to and recovery of the environment after a major disturbance. Rosenheim et al. [26] compiled stable carbon isotope data of sedimentary organic material across the Gulf of Mexico (GoM) prior to the DWH oil spill. While there was extensive ^13^C coverage in the GoM, there was little radiocarbon data so that baseline prior to the spill was underdetermined. Following the DWH oil spill, Chanton et al. [27] used radiocarbon to trace petrocarbon from the spill to the seafloor. Because of the lack of baseline ^14^C data in the GoM, Chanton et al. [27] used the nearly constant ^14^C values observed below the surface sediment layer to determine the pre-spill value. Surface sediment ^14^C values were more depleted than the underlying layers because of the addition of petrocarbon to the surface through sedimentation processes [27].

Sources of carbon to the sediments of the sGoM include marine production, which dominates the ^13^C signal [26], terrestrial carbon from rivers, hydrocarbon from oil spills, and hydrocarbons added from natural oil seeps, which occur throughout the GoM [28]. Seeps would be a source of ^13^C and ^14^C depleted organic material [29]. Several seep zones and oil slicks have been identified in the sGoM [28, 30, 31]. Many of these seeps and oil slicks were located in the Bay of Campeche and further offshore in the Campeche Knolls [28, 30, 31]. Holguin-Quiñones et al. [30] also identified seep locations along the coast between the Papaloapan River and the city of Tampico, as well as an area off the coast near the Mexico and United States border.

In this study we used stable carbon (δ^13^C, %C), nitrogen (δ^15^N, %N), and radiocarbon (Δ^14^C) analyses to examine sediment samples collected in the sGoM. Our first objective was to define the current baseline isotopic composition of the surface sediment of the sGoM and describe the isotopic spatial trends using isoscape maps. We examined the factors that might control the surface sediment baseline isotopic composition such as location (latitude, longitude), water depth, and distance from the coastline. This baseline information will be useful in determining inputs to the seafloor should oil spills occur in the future. Other isoscape maps produced for the GoM have focused on faunal δ^15^N, δ^13^C and δ^34^S on the West Florida Shelf in the northeast GoM [32, 33] and isocapes related to methyl-mercury in fish [34]. Peebles and Hollander [35] produced a δ^15^N isoscape map for the coastal areas of the entire GoM based on fish muscle.

Our second objective was to examine the region’s sedimentary temporal (depth) record for isotopic excursions in bulk organic material in an effort to determine the temporal preservation efficacy of such records. Such excursions could result from episodic terrestrial inputs, submarine oil seepage, or hydrocarbon spills, in particular the large Ixtoc 1 spill that occurred in 1979, some 40 years prior to our sampling. If excursions were found, we used ^210^Pb dating to determine the time that they occurred. A question we wanted to answer was if we could detect any evidence that the Ixtoc 1 record was preserved in bulk isotopes 36 years following the event.

## Materials and methods

**Fig 1:**
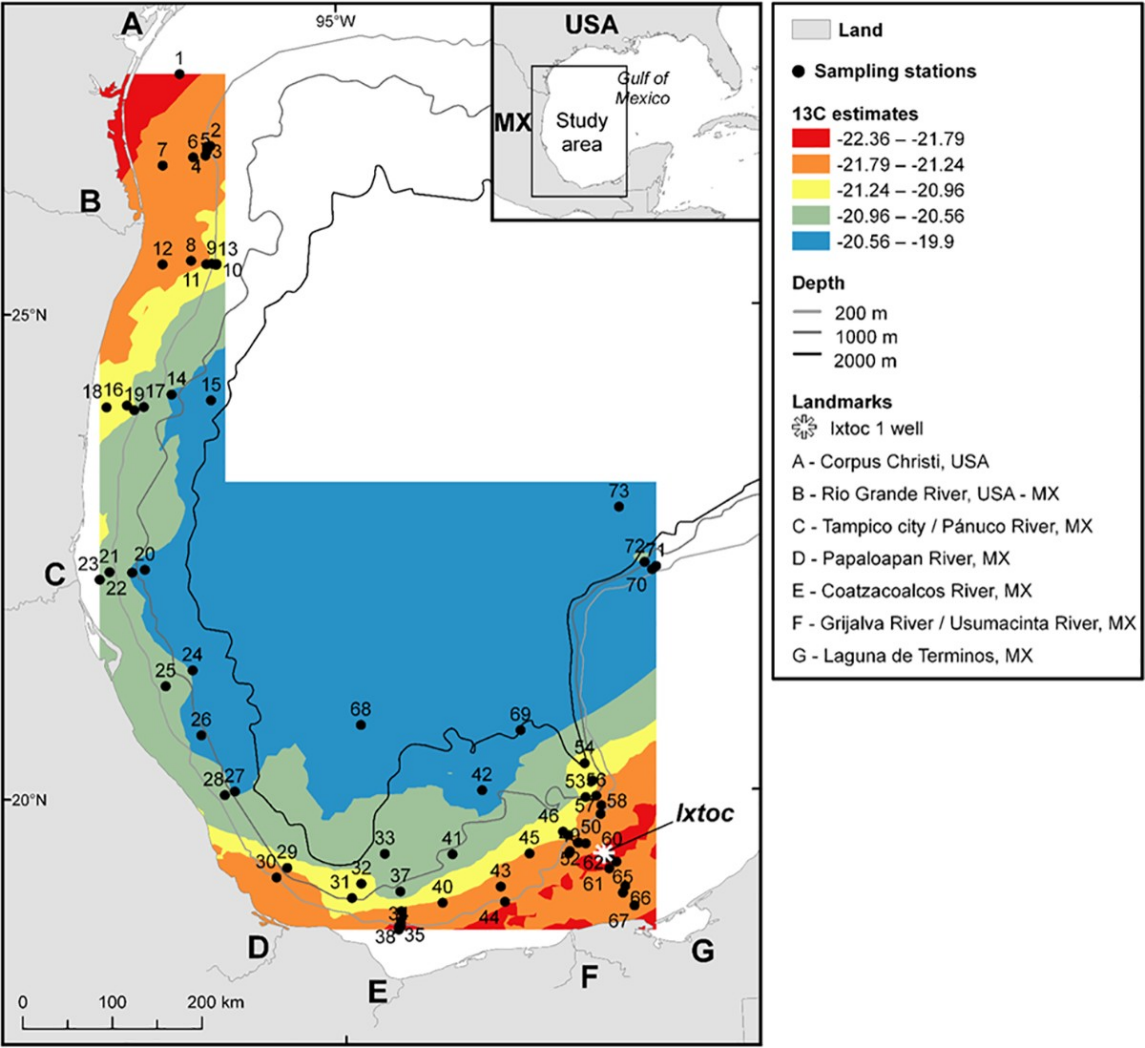
Map of the 73 sampling sites in the southern Gulf of Mexico with δ^13^C data. This map displays the spatial trend of δ^13^C values analyzed from all 73 surface sediment samples.

Ethics statement: No permissions were required because all sites were located in unprotected areas. This field study did not involve endangered or protected species. Sediment samples were collected in the sGoM (Fig 1) aboard the R/V Justo Sierra (in August 2015) and R/V Weatherbird II (in September 2015 and August 2016) using both multicore and a Shipek sediment grab sampler. The multicore collected 8 (R/V Weatherbird II) and 12 (R/V Justo Sierra) cores simultaneously and each core was used for a separate analysis (e.g. one core for bulk isotope analyses and one for short-lived radioisotope geochronology). Sampling sites were selected based on the Ixtoc 1 surface oil footprint and oil trajectories derived from satellite remote sensing data detected and quantified in Sun et al. [36]. In addition, samples from four cores collected in 2007, 2010 and 2011 were used in this study.

A total of 73 surface sediment samples were analyzed. These consisted of the 0–1 cm section of 41 multicore samples, the 2–3 cm section of three multicore samples collected in 2007, 2010 and 2011, where the 0–1 cm section was not available, and the estimated surface cm of 29 grab samples obtained with a Shipek sampler (S1 Table). However, not all isotope analyses (δ^13^C, Δ^14^C, δ^15^N, %C, and %N) were completed on all samples (e.g. only δ^13^C and Δ^14^C analysis were completed on the four multicore samples collected in 2007, 2010 and 2011, and three cores collected in 2016). Of the 44 multicore samples that were collected, 20 were chosen for analysis of selected sections down core (S1 Table). Cores collected for bulk isotope analyses were sectioned at 1 cm resolution at the University of South Florida’s College of Marine Science Paleo-Laboratory using an extrusion device [37]. Samples were treated with 10% HCl to remove carbonates, rinsed with DI water, freeze dried, and then ground with mortar and pestle prior to isotope analyses.

Stable carbon (δ^13^C, %C) and nitrogen (δ^15^N, %N) were measured using a Carlo-Erba elemental analyzer at the Duke Environmental Stable Isotope Laboratory. Sediment samples for radiocarbon (Δ^14^C) analyses were prepared at the National High Magnetic Field laboratory by combusting the samples in a muffle furnace at 850°C for four hours and then collecting the pure CO_2_ from the samples on a vacuum line using a series of cold traps to remove water vapor and non-condensable gases. Purified CO_2_ samples were then sent to the National Ocean Sciences Accelerator Mass Spectrometry (NOSAMS) facility or the University of Georgia (UGA) where the samples were prepared as graphic targets and analyzed by accelerator mass spectrometry [38].

Of the 20 cores where depth profiles were analyzed, two contained isotopic excursions from background values at depth. For these two sites, parallel cores from the multicore deployment were analyzed for ^210^Pb to determine the date of those excursion-containing horizons. Core IXNW1600 (site #68; Fig 1) was collected in September 2015 and sectioned at 2 mm (0–12 cm) and 5 mm (12–32 cm) resolution for analyses. Core E52 (Site #31; Fig 1) was collected in August 2011, split in half and sectioned at 1 cm resolution. Short-lived radioisotopes analyses were conducted by gamma spectrometry [39] on HPGe (High-Purity Germanium) Coaxial Planar Photon Detectors for total ^210^Pb (46.5 keV), ^214^Pb (295 keV and 352 keV) and ^214^Bi (609 keV). ^214^Pb and ^214^Bi activities were averaged as a proxy for ^226^Ra (or “supported” ^210^Pb, produced *in situ*; [40]). Supported ^210^Pb was subtracted from total ^210^Pb to calculate the “excess” ^210^Pb (^210^Pb_xs_), which is used for sediment age dating within the last ~100 years. ^210^Pb_xs_ was decay corrected to account for activity lost between core collection and sample analysis. To assess reproducibility of sediment records and geochronologies, samples from a companion core at site E52 (site #31) were analyzed for ^210^Pb through its radioactive descendant ^210^Po by alpha spectrometry at UNAM-Servicio Academico de Fechado, as described in Ruiz-Fernández and Hillaire-Marcel [41]. ^226^Ra was estimated using the lowest ^210^Po activity with no ^210^Pb_xs_ at 13 cm down core. Geochronologies were established by using the constant flux model (CF, also known as the constant rate of supply model CRS), as it is appropriate under conditions of varying accumulation rates, common in sedimentary systems [42–44], following the method described by Sanchez-Cabeza and Ruiz-Fernández [45].

Magnetic susceptibility analysis was also conducted on a companion core from site E52 (Site #31; Fig 1). Sediment aliquots of approximately 1.5 g were placed in a polyethylene tube (33 mm length, 8 mm diameter) and measured with a Bartington MS2 magnetic susceptibility meter coupled to a MSG2 frequency sensor. Replicate analysis of the Bartington-G039 calibration standard were used to evaluate accuracy (98%) and precision (variation coefficient = 4%) of the analysis.

To describe the baseline isotopic spatial trend in the sGoM, ArcGIS 10.7.1 was used to create isoscape maps for δ^13^C, Δ^14^C, δ^15^N, %C, and %N surface data. The kriging method was used to interpolate the raw values of each parameter. Depending on the nature of the raw data, data were log-transformed for non-normal distributions, de-trended, and accounted for anisotropy. The best model for each variable was chosen based on their root-mean-squared-standardized values (closest value to 1). Natural breaks (Jenks) were used to classify the interpolated values into categories. These models were computed using the Geostatistical Analyst Wizard.

## Results and discussion

### Isotopic baseline of the sGoM surface sediment

The first objective of this study was to determine baselines and trends in the isotopic composition of sedimentary organic matter across the sGoM. Surface sediment of all samples range in δ^13^C values from -22.4‰ to -19.9‰ (S1 Table) and have an average value of -21.2 ± 0.6‰ (standard deviation; n = 73), while Δ^14^C values range from -337.1‰ to -69.2‰ (S1 Table), and have an average of -164.6 ± 54.0‰ (n = 72). Surface sediment of all samples range in δ^15^N values from 2.8‰ to 7.2‰ (S1 Table), and have an average of 4.7 ± 0.9‰ (n = 66). The %C on a carbonate-free basis range in value from 0.65% to 3.89% (S1 Table), and average 1.45 ± 0.59% (n = 66), while %N range in value from 0.09% to 0.49% (S1 Table), with an average of 0.18 ± 0.07% (n = 66).

In a data set consisting of only surface sediments from the multicores (0–1 cm section), δ^13^C values range from -22.2‰ to -19.9‰ (S1 Table), with an average of -21.0 ± 0.6‰ (n = 41), while Δ^14^C range in value from -337.1‰ to -69.2‰ (S1 Table), with an average of -164.3 ± 63.1‰ (n = 41). δ^15^N range in value from 3.7‰ to 5.3‰ (S1 Table), and average 4.6 ± 0.4‰ (n = 37). %C range in value from 0.95% to 3.89% (S1 Table), with an average of 1.57 ± 0.58% (n = 37), while %N range in value from 0.14% to 0.49% (S1 Table), with an average of 0.20 ± 0.07% (n = 37). Due to the lack of differences, determined by T-test, in the average %C, Δ^14^C and δ^15^N values and only slight significant differences for δ^13^C and %N values (0.33‰ and 0.04%, respectively) when examining all surface sediment samples vs. only multicore (0–1 cm section) samples, we treated all samples as being representative of surficial sediment.

The isoscape map of δ^13^C surface sediment organic carbon values indicates the dominance of marine production with some terrestrial inputs in the nearshore. The map reveals more δ^13^C depleted areas in the south (Bay of Campeche) and towards the Laguna de Terminos (Fig 1 area G). This area is described as a transitional area with terrigenous clastic sediments found west of the Yucatan shelf, along the continental shelf, and biogenic carbonate deposits located on the Yucatan shelf [46, 47]. Depleted δ^13^C areas are observed along the coast, west-south-west of the Ixtoc 1 site (reference location) where three major rivers terminate into the sGoM (Grijalva-Usumacinta, Coatzacoalcos, Papaloapan; Fig 1 areas F, E & D). Depleted δ^13^C areas are also observed along the coast from north of Tampico (Fig 1 area C) to the Texas coastline by Corpus Christi (Fig 1 area A). More enriched δ^13^C areas are observed offshore, in deeper water. Soto and Escobar-Briones [48] determined that the food web of the inner shelf mostly receives estuarine input while the food web of the middle and outer shelf receives marine carbon sources.

**Fig 2:**
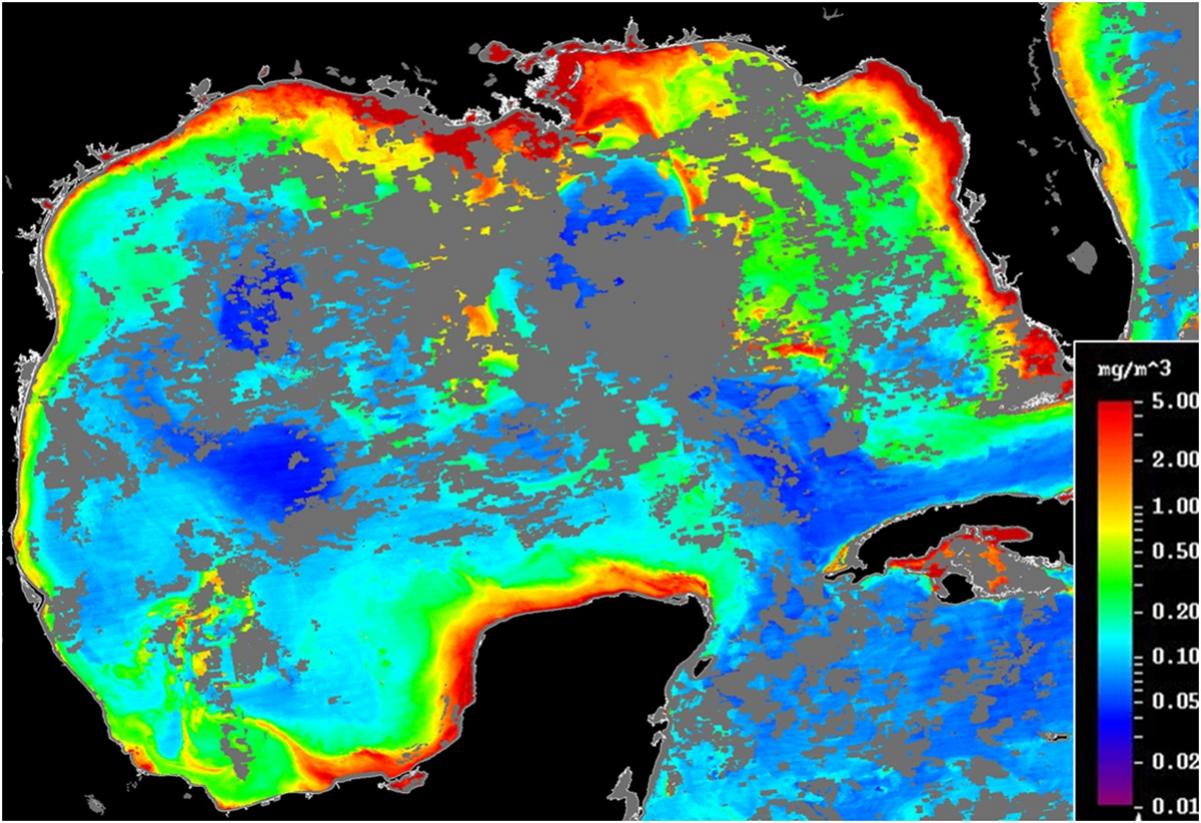
Map of chlorophyll (mg/m^3^) in the Gulf of Mexico from August 9 to 15, 2015. Reprinted from the USF optical oceanography lab under a CC BY license, with permission from Chuanmin Hu, original copyright 2015.

The δ^13^C isoscape map is similar to a map of chlorophyll concentration from a 2015 satellite imagery (Fig 2; [49]). This particular chlorophyll map is generally representative of chlorophyll at other times in the GoM. Higher concentrations of chlorophyll (0.8 to 5.0 mg/m^3^) are generally observed along the Mexican and Texas coast, with the highest concentrations (~3–5 mg/m^3^) occurring mostly from the Coatzacoalcos River to the Yucatan shelf, which encompasses the area of some of the most depleted δ^13^C sediments in our study area. The higher chlorophyll areas are likely related to terrestrial nutrient inputs. The surface sediment δ^13^C depletion is likely the result of depleted terrestrial carbon input from river flow or estuarine production added to marine production, rather than due to a δ^13^C induced response to enhanced rates of primary production in the open GoM. Enhanced primary production would likely result in the enrichment of δ^13^C of primary producers because at higher photosynthetic rates less isotopic fractionation occurs with respect to dissolved inorganic carbon during carbon fixation. In addition, enhanced nutrient concentration can result in larger phytoplankton cells which are also associated with increasing δ^13^C enrichment [50–53].

**Fig 3:**
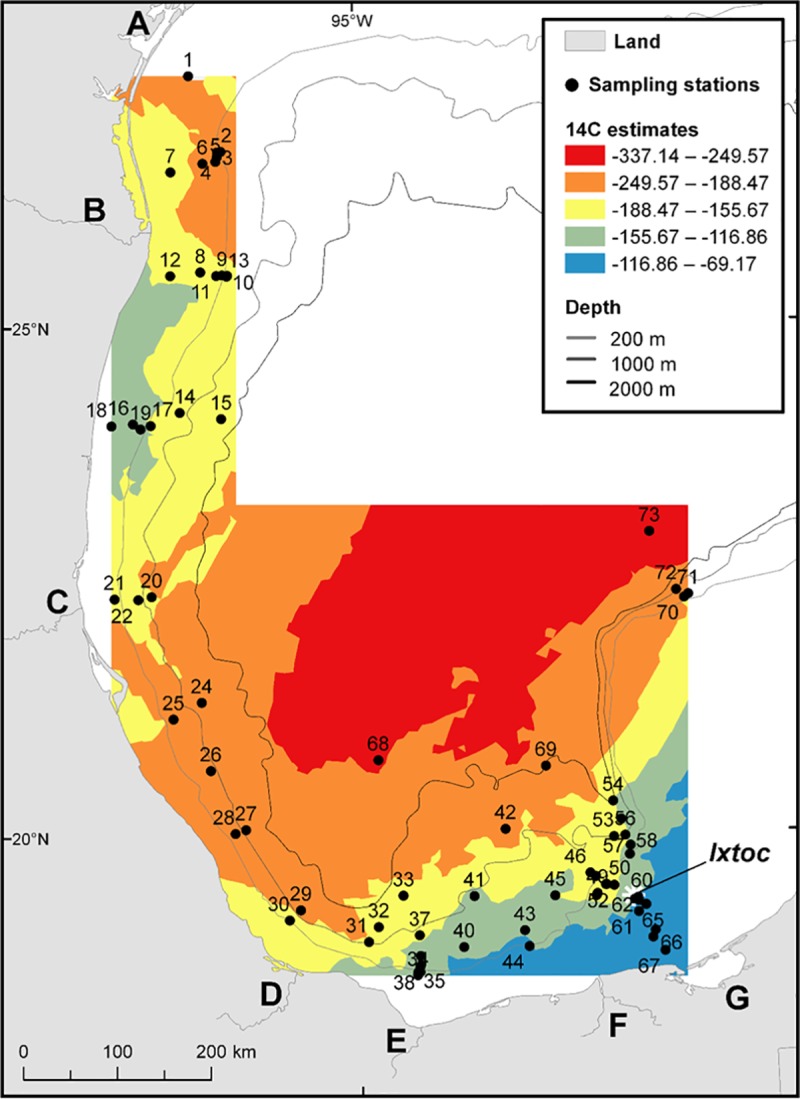
Map displaying the spatial trend of Δ^14^C values analyzed from 72 surface sediment samples. Landmark legend can be viewed on Fig 1.

A related spatial trend is observed on the Δ^14^C isoscape map (Fig 3). The area surrounding the Ixtoc 1 blowout site and towards the Laguna de Terminos consists of more modern, Δ^14^C enriched carbon, likely influenced by the Laguna de Terminos estuarine production (Fig 3 area G) and the Grijalva-Usumacinta Rivers (Fig 3 area F), while offshore areas are more depleted in Δ^14^C. The two most depleted sites (# 68; IXNW1600 and # 73; 2392) are located in water depths greater than 3000 m. These areas likely experience slower sedimentation rates and slower input rates of photosynthetically fixed marine material delivered from the surface. Areas of Δ^14^C depletion are also observed along the coastline between the Papaloapan River and Panuco River (Fig 3 area D and C) where relatively low surface water chlorophyll appears in the satellite image (Fig 2).

**Fig 4:**
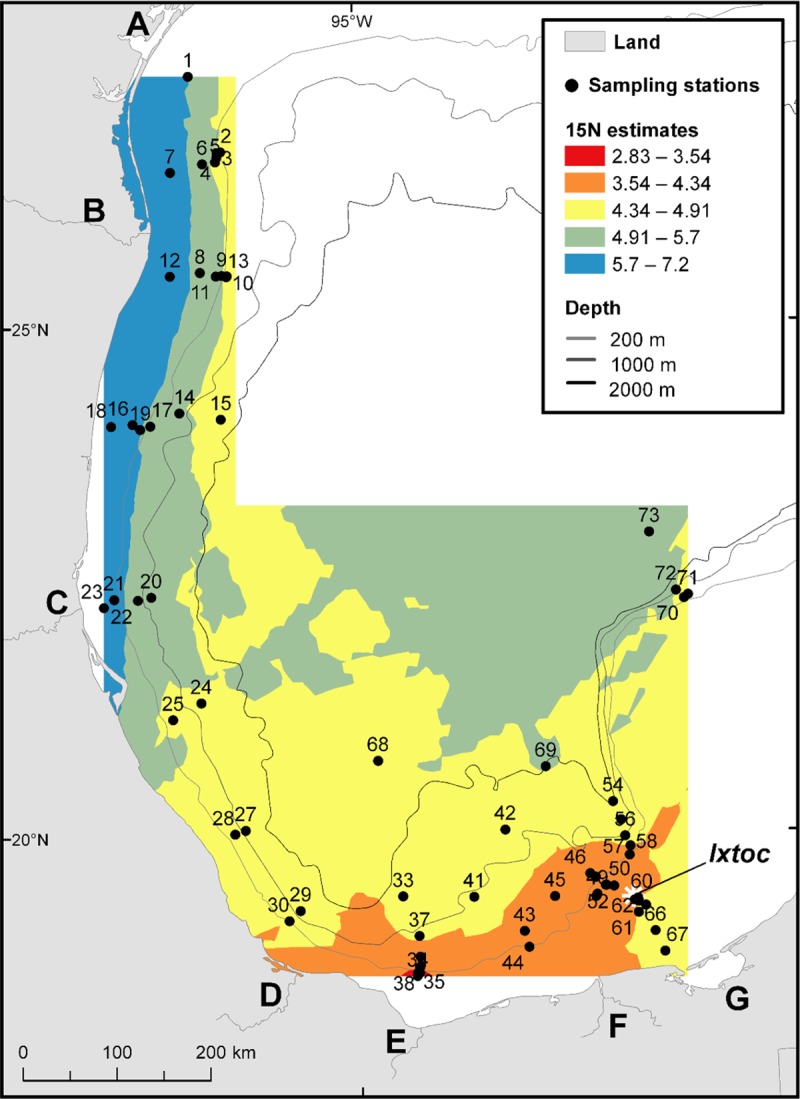
Map of the spatial trend of δ^15^N values analyzed from 66 surface sediment samples. Landmark legend can be viewed on Fig 1.

The isoscape map of δ^15^N in surface sediments reveals a latitudinal trend with more depleted δ^15^N areas observed around the Ixtoc 1 blowout site and the outputs of the three major rivers (Grijalva-Usumacinta, Coatzacoalcos, Papaloapan) and more enriched δ^15^N areas in the deeper waters, north of the Ixtoc 1 blowout site (Fig 4). A longitudinal gradient is also observed with more enriched δ^15^N areas along the northern Mexican to south Texas coastline and more depleted areas offshore. A similar pattern is reported in Peebles and Hollander [35]’s large scale study of δ^15^N from red snapper muscle, where δ^15^N values are lowest in the southeastern portions of the sGoM and increase towards the northwest. More depleted δ^15^N values can be associated with terrestrial organic material [54–56], but this is not always the case [57].

**Fig 5:**
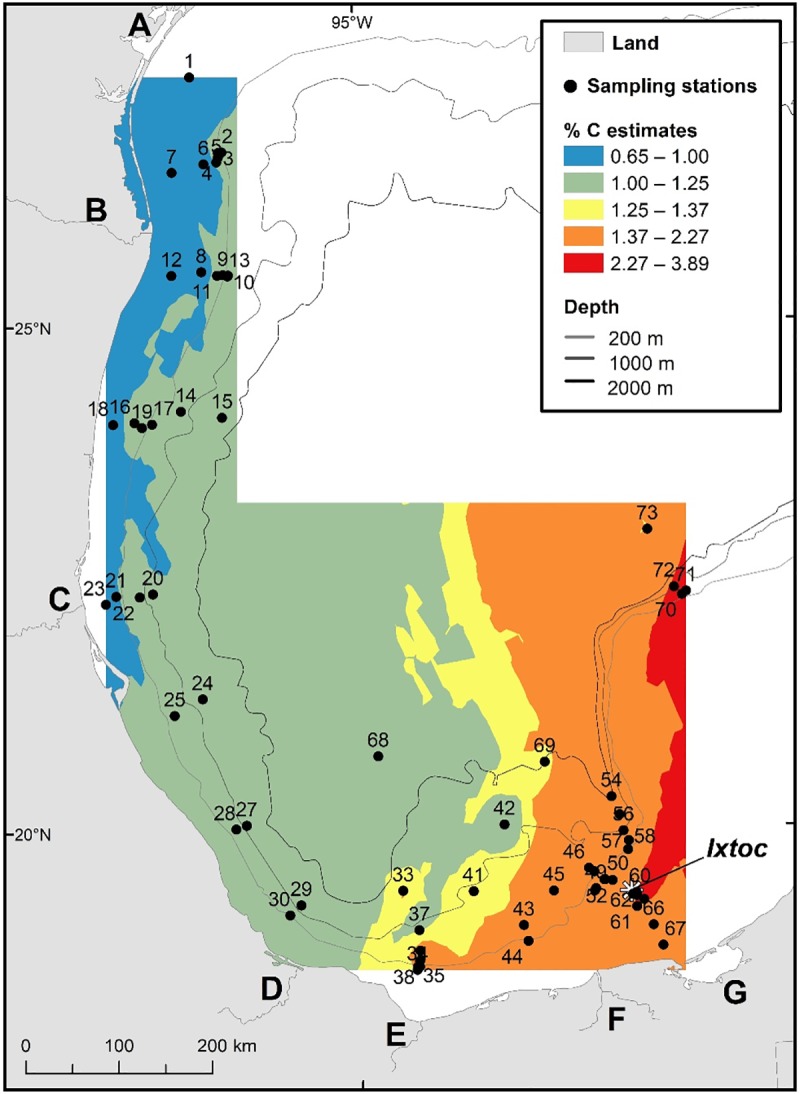
Map of the spatial trend of %C values analyzed from 66 surface sediment samples. Landmark legend can be viewed on Fig 1.

**Fig 6:**
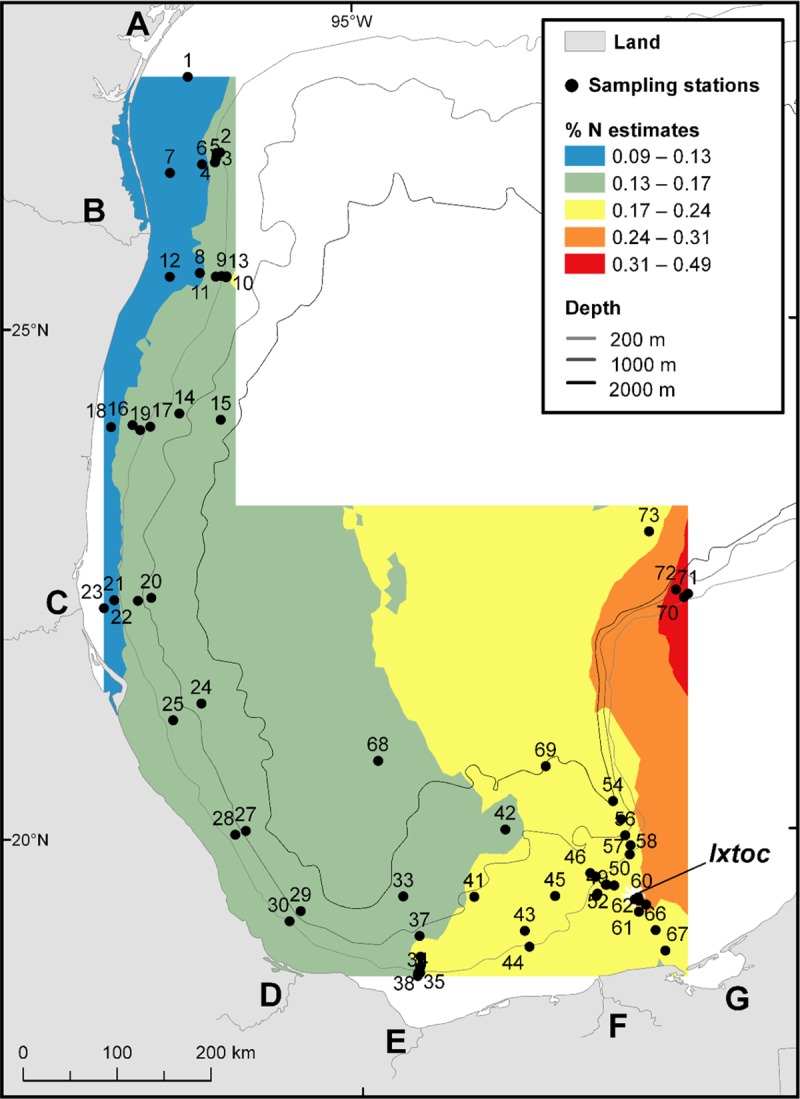
Map of the spatial trend of %N values analyzed from 66 surface sediment samples. Landmark legend can be viewed on Fig 1.

Both the %C and %N isoscape maps reveal a longitudinal trend with higher percentages observed in the eastern part of the sampling region, moving towards lower percentages to the west (Figs 5 and 6), consistent with higher productivity in the east. Large areas of high chlorophyll can be seen on the Yucatan shelf in satellite imagery (Fig 2). Areas of upwelling have been described on the Campeche Bank [3], along the Yucatan Strait, and could contribute to higher productivity and greater input of organic matter to the seafloor. Escobar-Briones and Garcia-Villalobos [58] reported relatively low %C and %N values in the continental slope sediments (1,000–2,800 m) and the highest values in the abyssal plain sediments (>3,600 m).

### Correlations with surface sediment isotopic composition

**Fig 7:**
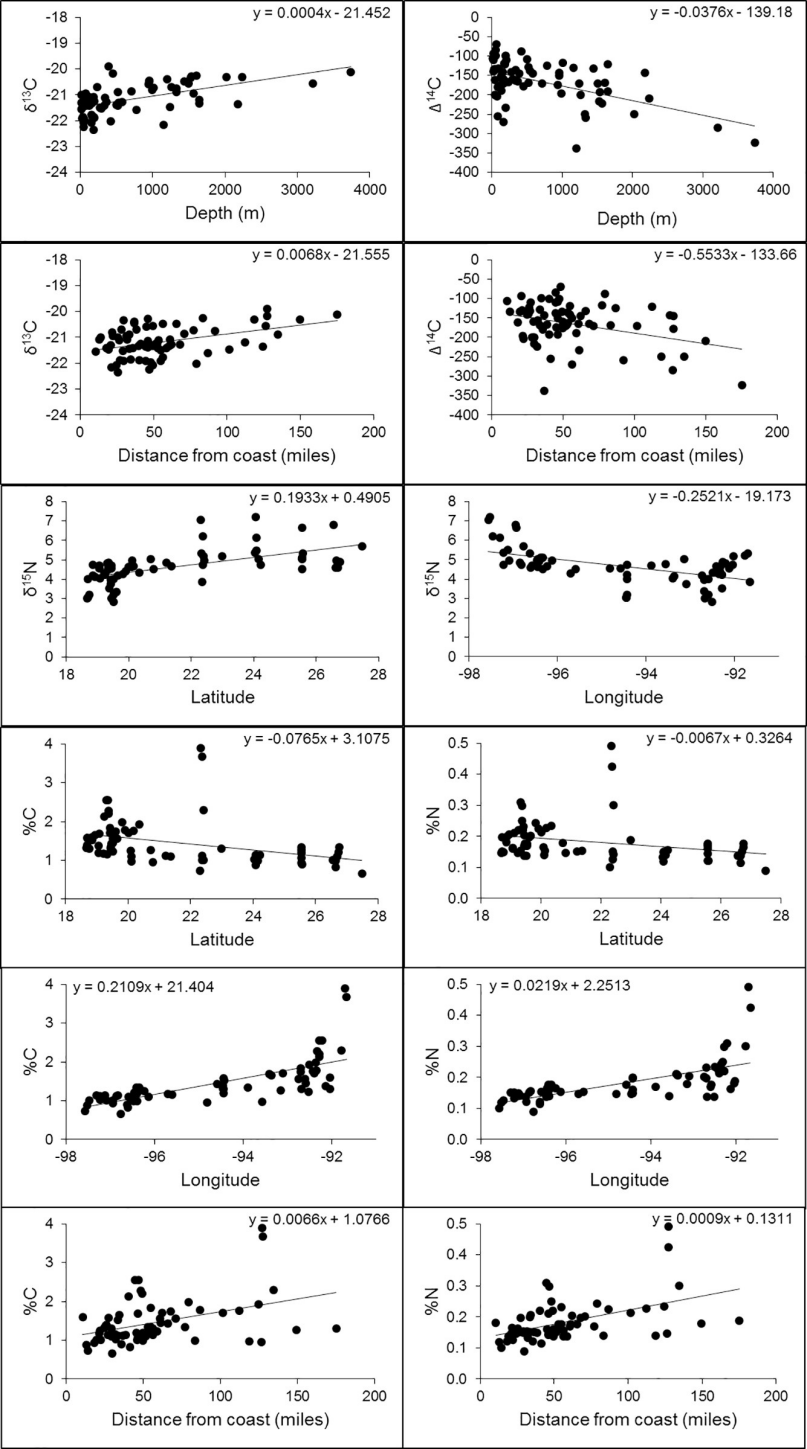
Trends of surface sediment isotopic data vs latitude, longitude, water depth, and distance from coast. Only the significant correlations are shown.

There are no latitudinal or longitudinal correlations with the surface sediment δ^13^C or Δ^14^C values (Table 1). A significant correlation is observed between water depth and the δ^13^C and Δ^14^C values of the surface sediment (Table 1 and Fig 7), where more depleted δ^13^C sites are located at shallow water depth sites and more depleted Δ^14^C sites are located in deeper sampling sites (Figs 1 and 3). There is also a significant correlation between distance from the coastline and the δ^13^C and Δ^14^C values (Table 1 and Fig 7), where more depleted δ^13^C sites are located closer to the coastline and more depleted Δ^14^C sites are located offshore (Figs 1 and 3). Of course, distance from the coast and water depth are highly correlated (r = 0.66).

**Table 1:** Regression of surface sediment isotopic data vs latitude, longitude, water depth, and distance from coastline.

	r	n	p
δ^13^C vs latitude	0.1761	73	0.136
δ^13^C vs longitude	0.0510	73	0.666
**δ**^**13**^**C vs depth**	**0.5612**	**73**	**<0.001**
**δ**^**13**^**C vs distance from coastline**	**0.4226**	**73**	**<0.001**
Δ^14^C vs latitude	0.1764	72	0.138
Δ^14^C vs longitude	0.1646	72	0.167
**Δ**^**14**^**C vs depth**	**0.5403**	**72**	**<0.001**
**Δ**^**14**^**C vs distance from coastline**	**0.3593**	**72**	**0.002**
**δ**^**15**^**N vs latitude**	**0.5925**	**66**	**<0.001**
**δ**^**15**^**N vs longitude**	**0.5609**	**66**	**<0.001**
δ^15^N vs depth	0.0047	66	0.97
δ^15^N vs distance from coastline	0.1364	66	0.27
**%C vs latitude**	**0.3597**	**66**	**0.003**
**%C vs longitude**	**0.7204**	**66**	**<0.001**
%C vs depth	0.0954	66	0.45
**%C vs distance from coastline**	**0.4052**	**66**	**<0.001**
**%N vs latitude**	**0.2771**	**66**	**0.024**
**%N vs longitude**	**0.6612**	**66**	**<0.001**
%N vs depth	0.0069	66	0.96
**%N distance from coastline**	**0.4892**	**66**	**<0.001**

Those with significant correlations are highlighted in bold.

There are significant correlations between latitude and the surface sediment values of δ^15^N, %C, and %N, and between longitude and the surface sediment values of δ^15^N, %C and %N (Table 1 and Fig 7). However, there are no significant correlations between water depth and surface sediment values of δ^15^N (Table 1). There is also no significant correlation between distance from the coastline and the surface sediment value of δ^15^N, but there are significant correlations between distance from the coastline and the surface sediment values of %C and %N (Table 1 and Fig 7).

### Isotopic excursions relative to baseline at depth in sediments

**Fig 8:**
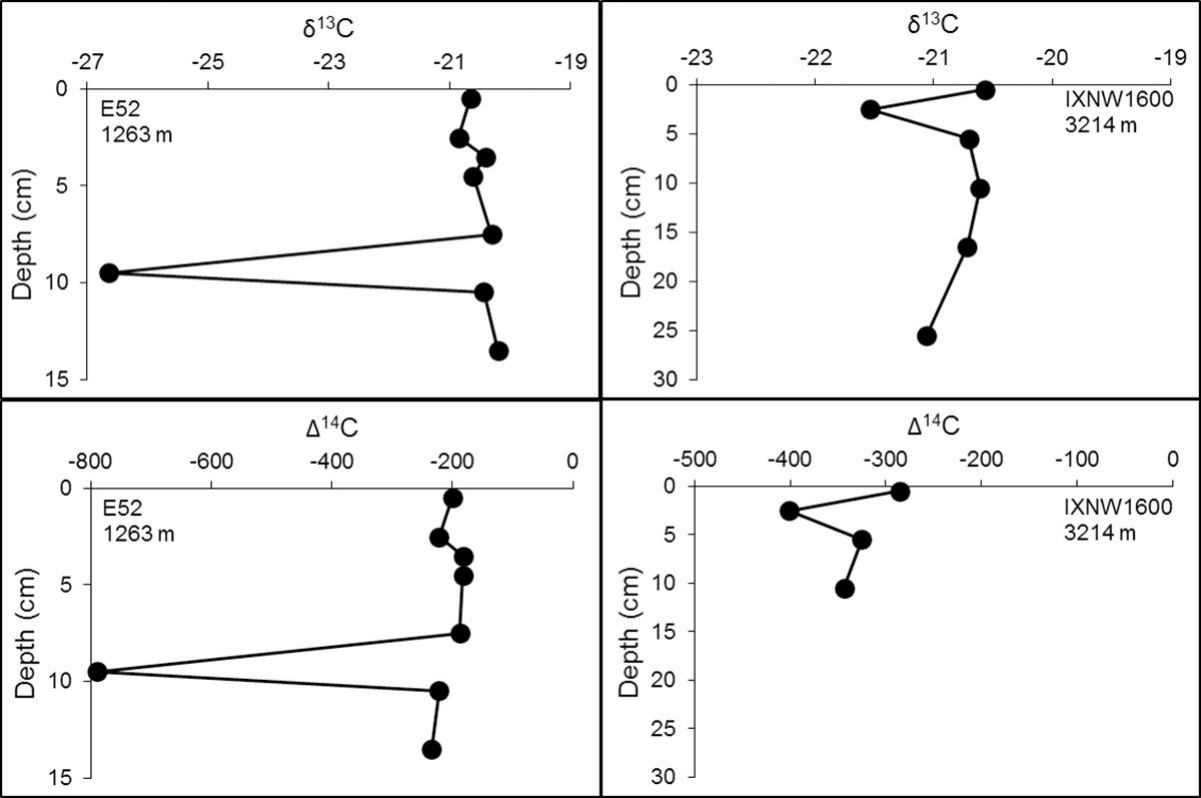
Isotopic (δ^13^C and Δ^14^C) profiles displaying depleted isotope values at a specific depth. Water depth at which the core was collected at is below core name on plots. The remaining core profiles can be seen in S1 Fig.

Our second objective was to examine the region for past influences of hydrocarbon deposition in the sedimentary temporal (depth) record that might be associated with hydrocarbon recovery, spillage and seepage, as was found in the northern Gulf of Mexico (nGoM) following the DWH oil spill in 2010. We wanted to determine if there was any remaining evidence consistent with the Ixtoc 1 event that might still be preserved at depth in the isotopic record of the bulk sediments. A total of 20 cores were selected and analyzed as a function of core depth to look for organic carbon horizons that were depleted in δ^13^C and Δ^14^C, consistent with the addition of petrocarbon at the times those layers were deposited [27, 59] (Fig 8; S1 Fig). In the nGoM, depleted δ^13^C and Δ^14^C values indicative of petrocarbon residue from a sedimentation event were observed in cores collected in 2010, shortly after the DWH oil spill. In these nGoM cores, the 0–1 cm layer was considerably more depleted in Δ^14^C compared to layers below it [27]. However, the preservation of such layers for extended periods of time in the sedimentary record is uncertain [59].

Of the 20 cores we examined, only two had negative isotopic excursions at depth (Fig 8). Core E52 (site #31; Figs 1 and 3) was collected in 2011 just offshore between the rivers, Papaloapan and Coatzacoalcos. Negative isotope excursions appear at the 9–10 cm horizon in both C isotope plots (Fig 8). The δ^13^C value of the 9–10 cm section is -26.6‰, while the δ^13^C values of the other sections examined range from -20.8‰ to -20.2‰. The Δ^14^C value of the 9–10 cm section is -789.5‰, while the Δ^14^C values of the other sections range from -234.7‰ to -182.5‰. The large difference in values in the 9–10 cm section compared to the values at the other sections examined and the fact that these large depletions are seen for both the Δ^14^C and δ^13^C data at the same section, could be explained by either a petrocarbon source or a terrestrially derived source.

**Fig 9:**
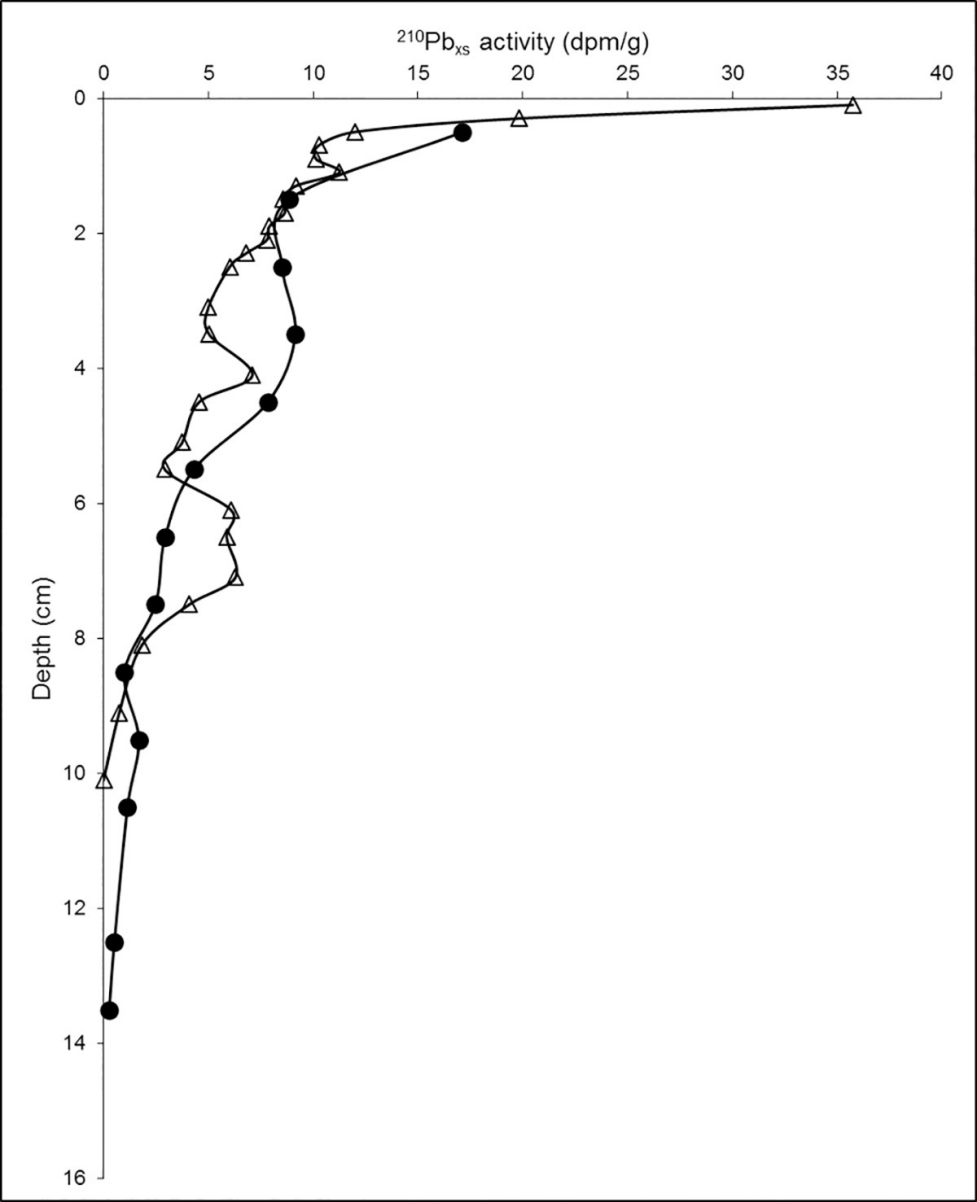
Excess ^210^Pb_xs_ in sediments as a function of depth. Core E52 (filled circles) and core IXNW1600 (open triangles).

The ^210^Pb_xs_ geochronologies of Core E52 collected at 1263 m depth (Fig 9 and Table 2) were comparable for both analytical methods (gamma and alpha spectrometry) and do not indicate that the layer containing the isotopic excursion was deposited at the time of the Ixtoc 1 oil spill event. The 9–10 cm section appears to coincide with the early part of the 20^th^ century (Table 2). According to the ^210^Pb_xs_ chronology, any evidence of the Ixtoc 1 oil spill event would have been recorded around the 3–4 cm section, where our δ^13^C and Δ^14^C results do not suggest the presence of petrocarbon residues. If Ixtoc 1 oil was deposited at this time, then the lack of an isotopic signal in the bulk organic material at 3–4 cm must be due to degradation of petrocarbon following its deposition [59–61].

**Table 2:** Application of the constant flux model for cores E52 (site 31) and IXNW1600 (site 68).

E52			IXNW1600		
Depth (cm)	Mean year	± Uncertainty (years)	Depth (cm)	Mean year	± Uncertainty (years)
0.5	2005.9	0.1	0.1	2014.7	3.9
1.5	1997.6	0.2	0.3	2013.1	3.9
2.5	1990.9	0.3	0.5	2011.6	4.0
3.5	1981.4	0.5	0.7	2010.4	4.1
4.5	1970.2	0.7	0.9	2009.2	4.1
5.5	1960.4	1.0	1.1	2007.8	4.2
6.5	1951.5	1.2	1.3	2006.4	4.3
7.5	1941.0	1.5	1.5	2005.1	4.4
8.5	1928.6	2.1	1.7	2003.8	4.4
9.5	1913.2	3.0	1.9	2002.4	4.5
10.5	1893.5	4.7	2.1	2001.0	4.6
11.5	1866.4	8.7	2.3	1999.7	4.7
			2.5	1998.5	4.8
			3.1	1995.0	5.0
			3.5	1992.6	5.2
			4.1	1987.6	5.7
			4.5	1983.9	6.1
			5.1	1979.5	6.6
			5.5	1976.7	6.9
			6.1	1970.1	8.0
			6.5	1962.6	9.6
			7.1	1946.1	14.9
			7.5	1931.0	22.9
			8.1	1907.3	45.4

**Fig 10:**
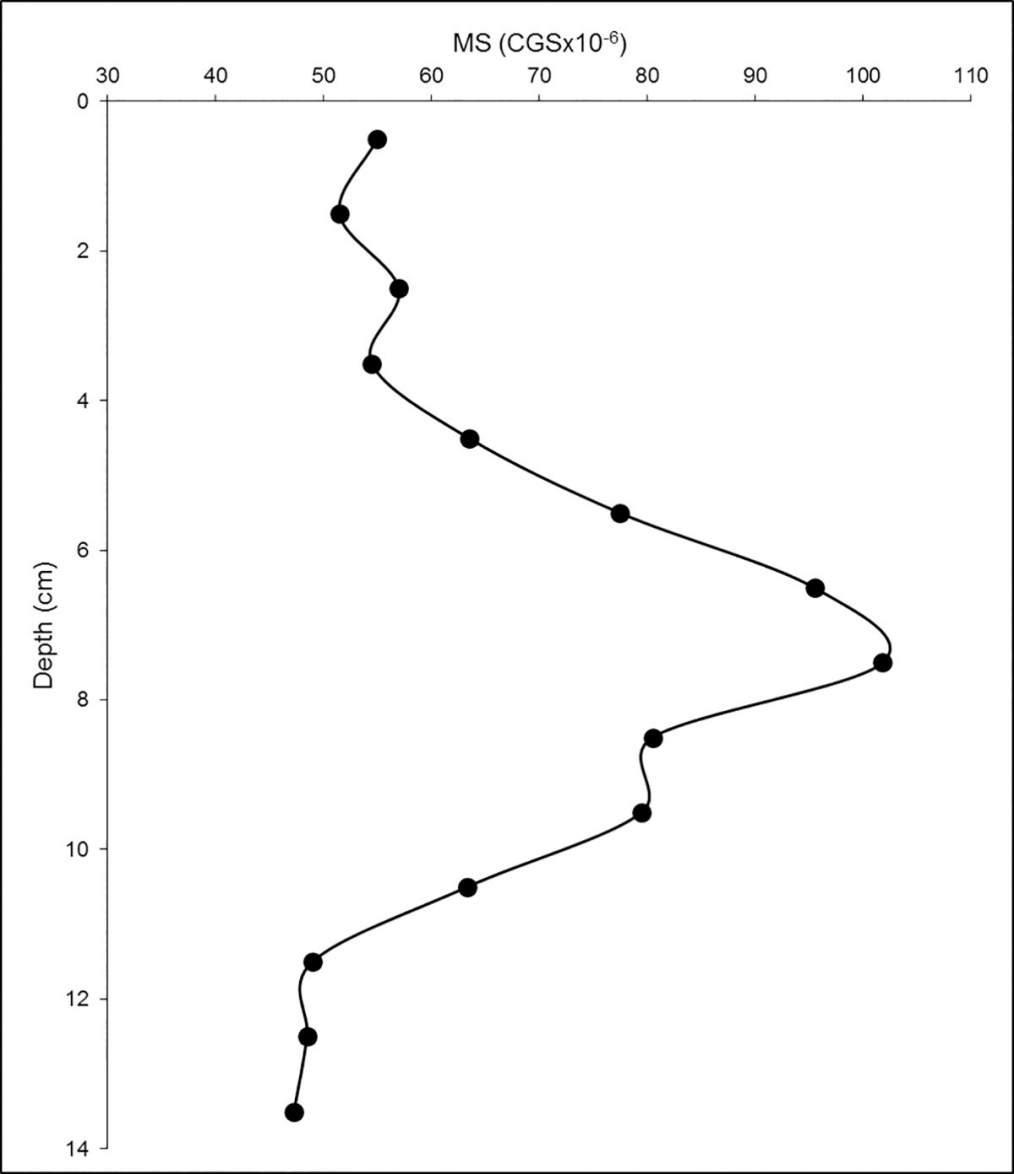
Magnetic susceptibility (MS) as a function of depth in core E52.

However, a depositional event during the early 1900’s apparently resulted in a large depletion in δ^13^C and Δ^14^C values at the 9–10 cm section of the E52 (site #31) core, which remained preserved for 80 plus years. Terrigenous inputs can also cause depletions in δ^13^C and Δ^14^C values. Magnetic susceptibility ranged from 47.3 to 101.8 CGS x 10^−6^, with a large peak observed between 4 and 11 cm depth (Fig 10). Magnetic susceptibility is an indicator for deposition of detrital constituents into the marine environment [62]. Ellwood et al. [62] identified a sediment detrital pathway in the sGoM from the Veracruz Tongue (located in close proximity to the Papaloapan River and site E52) to the southern margin of the Sigsbee Abyssal Plain.

Core IXNW1600 (site #68; Figs 1 and 3), collected in September 2015, is located in waters greater than 3000 m. Isotopic depletion in core IXNW1600 is observed at the 2–3 cm section where the δ^13^C value is -21.5‰, while the δ^13^C value of the other sections examined in the core range from -21.1‰ to -20.6‰. The Δ^14^C value of the 2–3 cm section is -401.1‰, while the Δ^14^C value of the other sections range from -343.7‰ to -285.3‰. Again, ^210^Pb_xs_ dating does not support petrocarbon deposition from the Ixtoc 1 oil spill event (Fig 9 and Table 2) as the 2–3 cm section coincides with the mid 1990s and early 2000s. The ^210^Pb_xs_ geochronology suggests that we might expect to see evidence of the Ixtoc 1 oil spill event around 5 cm, however, the δ^13^C and Δ^14^C values at the 5–6 cm section in our study do not suggest preservation of petrocarbon residues. This core also happens to be located within the sediment detrital pathway identified by Ellwood et al. [62], with the IXNW1600 site being located in the southern margin of the Sigsbee Abyssal Plain.

**Fig 11:**
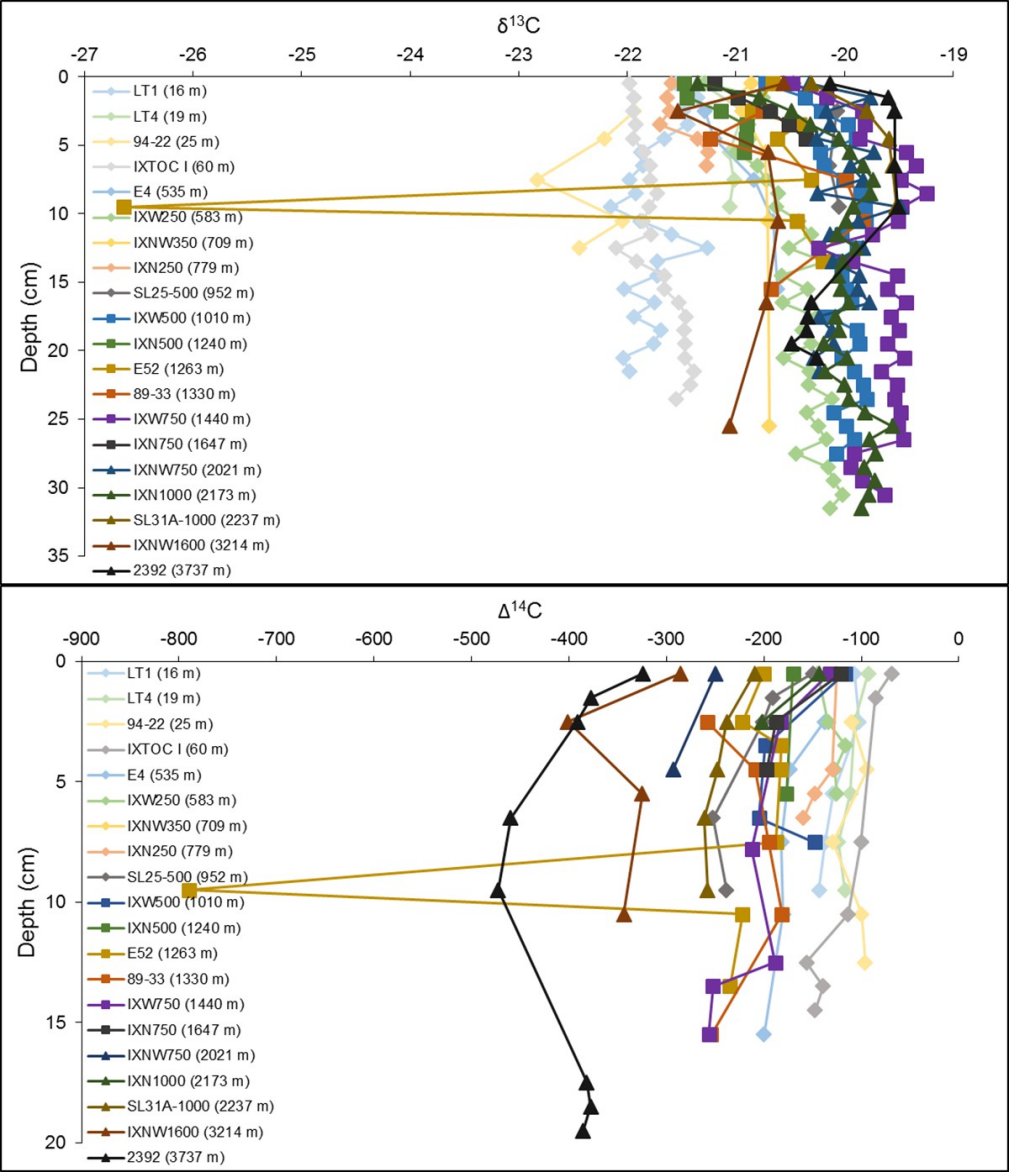
**Core profiles of δ**^**13**^**C (top) and Δ**^**14**^**C (bottom) for the 20 cores examined.** Sampling sites listed in the legend are ordered from shallowest depth (lighter colors, diamonds) to deepest water depth (darker colors, squares, triangles) and range from 16 to 3737 m.

No other cores had depleted horizons indicative of a petrocarbon deposition event (Fig 11 and S1 Fig). The dominant trend in the core profiles is a relationship with water depth. Cores collected in shallower waters generally are more depleted in δ^13^C throughout the core than those collected in the deeper waters of the sampling area. The opposite pattern occurs for Δ^14^C where cores collected in shallower waters are generally more enriched throughout the core than those collected in deeper waters (Fig 11).

The warm waters in the shallow depths where the Ixtoc 1 oil spill occurred appears to have resulted in the biodegradation of any petrocarbon which might have been deposited at the levels necessary for us to observe in the bulk sedimentary organic carbon isotopic values in samples collected in 2011 and 2015–2016. Some tar balls can still be found in remote coastal areas of Mexico [10], but we found no evidence that significant quantities of petrocarbon are preserved on the seafloor. Complex habitats, like mangroves and salt marshes, retain oil much longer [63], and oil buried in the more rapidly accumulating sediment of nearshore zones can take longer to degrade due to more anoxic conditions [46]. Other methods being applied to the study area may be more effective in revealing preserved evidence of an Ixtoc 1 MOSSFA event, including microbiology analyses, biomarkers (e.g. hopanes, steranes and diasteranes), and benthic foraminifera assemblages and their isotopic composition [64–67].

Overholt et al. [68] examined the benthic marine microbial community of the GoM and found no evidence of a disturbed microbial community in sediment cores collected in the sGoM due to the Ixtoc 1 oil spill. However, they stated that the nGoM microbial community had returned to baseline conditions two years after the DWH oil spill, so this would be expected. Lincoln et al. [69] used biomarker approaches to identify evidence of Ixtoc 1 oil in the sGoM cores, while Schwing et al. [65] used a multi-proxy approach involving short-lived radioisotopes (^210^Pb_xs_) and benthic foraminifera stable isotopes (δ^13^C _CaCO3_). These approaches identified preserved evidence of petrocarbon sources consistent with the Ixtoc 1 event in the sedimentary record at site IXW250 (site #45; Fig 1). We found no evidence of Ixtoc 1 oil remaining in the sGoM sediments using bulk organic δ^13^C and Δ^14^C analyses.

Consistent with our results, studies of seafloor petrocarbon resulting from the DWH oil spill in the nGoM indicate that bulk isotopes would not be a robust indicator for identifying oil deposited in 1979–80. Rogers et al. [59] observed a recovery of Δ^14^C composition in sediments in the nGoM in ~5 years. Stout et al. [60] observed considerably less hopane biomarker for DWH oil in surface/near surface sediments collected in 2014 compared to those collected in 2010/2011. Chanton et al. [70] observed a recovery in suspended sinking particles three years after the DWH oil spill at sites near the well using Δ^14^C analyses. Rogers et al. [71] observed recovery in suspended particulates after four years.

In addition, despite the presence of seep sites within the sGoM, the isotopic shifts towards δ^13^C and Δ^14^C depletion typically associated with these features [27] are not evident in our data set with the exception of the two isotopic excursions observed, which appear to be due to terrestrially derived sources. It would appear that the zones of seep influence are relatively localized.

## Conclusion

As oil exploration continues around the world, it has become increasingly important to understand and characterize the ecosystems in which these extractions take place. Establishing a baseline of environmental conditions is useful for accessing the potential impacts and recovery of the environment after a disturbance. Isotopic tracers offer a unique perspective on quantifying these impacts because they reveal the quantity of altered and unaltered oil-residue. In our study, we found that the δ^13^C and Δ^14^C isotopic spatial trends were related to depth and distance from the coastline, while latitude and longitude were related to the δ^15^N, %C and %N composition of the sediments. We were also unable to detect evidence of an oil deposition in the isotopic composition in bulk sediments in the sGoM, 36 years after the Ixtoc 1 oil spill.

The results of this paper are consistent with the findings of Rogers et al. [59], who reported that the depletion of bulk radiocarbon values that were observed with the sedimentation of oil-residues in the nGoM immediately after the 2010 DWH spill [27] persisted for a period of about five years post-spill. Thus, bulk radiocarbon analysis of sediments following an oil spill is extremely useful and allows for the determination of instantaneous inputs of petroleum-derived carbon to the seafloor, especially if one has a pre-spill surface sediment isoscape as provided by this paper. The advantage of radiocarbon for deriving these inputs is two-fold. First, following one spill, it will reset back baseline values in years allowing for quantification of subsequent spills. Second, radiocarbon tracking has the capability to yield estimates of both petroleum and transformed petroleum-material that might not be readily identifiable by other approaches. For example, during the 2010 DWH oil spill, a deep water plume at 1000–1200 m depth carried some 30% of the material issued from the broken well [72]. This plume was colonized by a succession of microbes [73–75] which produced floc consisting of carbohydrates and cell biomass which were derived from the hydrocarbon substrate [76]. These same microbes were identified on the seafloor [77]. The extent to which their biomass and floc contributed to seafloor petrocarbon was readily identifiable from radiocarbon tracing of the bulk sediments. Similarly, the large quantity of methane that was issued from the broken well was rapidly oxidized by methanotrophic bacteria [78, 79], which are very efficient at producing cell biomass. This allowed methane to enter the particulate phase and subsequently the GoM food web [80–82]. Radiocarbon tracing allowed the identification of this pathway, when tracers more specific to hydrocarbons, would not have identified the material as such.

## Supporting information

S1 Table List of samples used for surface sediment analyses and the isotope results. Section (cm) indicates the core section used in analyses for samples collected by multicore. Where sediment grabs (listed as grab under column heading, Sample equipment) were used to collect samples, n/a is indicated for section. Samples collected by multicore and analyzed down core, in addition to surface sections, are highlighted in bold.
(PDF)

S1 Fig Isotopic (δ^13^C and Δ^14^C) core profiles for the remaining 18 cores examined in the southern Gulf of Mexico. Cores are ordered from shallowest water depth to deepest water depth.
(PDF)
