# Supplementary material for: The southern Gulf of Mexico: A baseline radiocarbon isoscape of surface sediments and isotopic excursions at depth
Source: PLoS One. 2020 Apr 15;15(4):e0231678. doi: 10.1371/journal.pone.0231678 (PMC7159241; doi:10.1371/journal.pone.0231678)
Supplement: S1 Table — Section (cm) indicates the core section used in analyses for samples collected by multicore. Where sediment grabs (listed as grab under column heading, Sample equipment) were used to collect samples, n/a is indicated for section. Samples collected by multicore and analyzed down core, in addition to surface sections, are highlighted in bold. (PDF) [file pone.0231678.s001.pdf]

**S1 Table. List of samples used for surface sediment analyses and the isotope results.** Section (cm) indicates the core section used in analyses for samples collected by multicore. Where sediment grabs (listed as grab under column heading, Sample equipment) were used to collect samples, n/a is indicated for section. Samples collected by multicore and analyzed down core, in addition to surface sections, are highlighted in bold.

| **Sample ID** | **Section (cm)** | **Site #** | **Sample equipment** | **Collection date** | **Latitude** | **Longitude** | **Depth** | **$\delta^{13}C$** | **$\delta^{15}N$** | **%C** | **%N** | **$\Delta^{14}C$** |
|---|---|---|---|---|---|---|---|---|---|---|---|---|
| SL26A-250 | 0-1 | 25 | multicore | 7/30/2015 | 21.2103 | -96.8556 | 518 | -20.88 | 4.85 | 1.11 | 0.15 | -169.67 |
| **IXNW1600** | 0-1 | 68 | multicore | 7/31/2015 | 20.8066 | -94.8136 | 3214 | -20.56 | 4.54 | 0.95 | 0.15 | -285.34 |
| SL26A-750 | 0-1 | 24 | multicore | 7/31/2015 | 21.3765 | -96.5746 | 1499 | -20.56 | 4.68 | 1.09 | 0.15 | -171.36 |
| SL33-200 | 0-1 | 70 | multicore | 8/1/2015 | 22.3319 | -91.7015 | 391 | -19.90 | 5.33 | 3.89 | 0.49 | -145.58 |
| SL33-675 | 0-1 | 72 | multicore | 8/1/2015 | 22.4102 | -91.7780 | 1326 | -20.88 | 5.20 | 2.29 | 0.30 | -249.57 |
| **2392** | 0-1 | 73 | multicore | 8/1/2015 | 22.9895 | -92.0269 | 3737 | -20.13 | 5.19 | 1.31 | 0.19 | -323.87 |
| **IXN1000** | 0-1 | 54 | multicore | 8/2/2015 | 20.3525 | -92.4946 | 2173 | -21.35 | 4.34 | 1.93 | 0.23 | -143.10 |
| **SL31A-1000** | 0-1 | 69 | multicore | 8/2/2015 | 20.7184 | -93.1492 | 2237 | -20.30 | 5.03 | 1.27 | 0.18 | -209.57 |
| **IXTOC1** | 0-1 | 59 | multicore | 8/3/2015 | 19.3701 | -92.3172 | 60 | -21.98 | 4.65 | 2.28 | 0.25 | -69.17 |
| IXN100 | 0-1 | 57 | multicore | 8/3/2015 | 19.8175 | -92.3484 | 421 | -22.03 | 4.27 | 1.98 | 0.24 | -87.94 |
| **IXN750** | 0-1 | 55 | multicore | 8/3/2015 | 20.1701 | -92.4200 | 1647 | -21.20 | 4.66 | 1.77 | 0.23 | -121.12 |
| SL31-100 | 0-1 | 50 | multicore | 8/3/2015 | 19.5255 | -92.5857 | 190 | -21.07 | 4.01 | 1.45 | 0.18 | -233.74 |
| Abkatun | 0-1 | 63 | multicore | 8/3/2015 | 19.3143 | -92.2080 | 50 | -21.37 | 4.82 | 2.56 | 0.31 | -85.11 |
| **IXN250** | 0-1 | 58 | multicore | 8/3/2015 | 19.9080 | -92.3373 | 779 | -21.59 | 4.42 | 1.78 | 0.22 | -125.62 |
| **IXN500** | 0-1 | 56 | multicore | 8/3/2015 | 20.0089 | -92.3865 | 1240 | -21.47 | 4.64 | 1.71 | 0.21 | -170.17 |
| LT3 | 0-1 | 62 | multicore | 8/3/2015 | 19.3568 | -92.2759 | 51 | -22.24 | 4.87 | 2.54 | 0.30 | -100.01 |
| **LT1** | 0-1 | 67 | multicore | 8/4/2015 | 18.8484 | -92.0371 | 16 | -21.55 | 4.74 | 1.60 | 0.18 | -107.17 |
| IXW100 | 0-1 | 47 | multicore | 8/4/2015 | 19.4187 | -92.6894 | 179 | -21.91 | 3.97 | 1.84 | 0.23 | -119.05 |
| LT2 | 0-1 | 66 | multicore | 8/4/2015 | 19.0564 | -92.1245 | 21 | -21.00 | 4.56 | 1.38 | 0.16 | -139.86 |
| **IXNW350** | 0-1 | 46 | multicore | 8/4/2015 | 19.6457 | -92.7421 | 709 | -20.86 | 4.19 | 1.55 | 0.20 | -170.63 |
| **IXW750** | 0-1 | 33 | multicore | 8/5/2015 | 19.4600 | -94.5849 | 1440 | -20.48 | 4.54 | 1.43 | 0.18 | -132.30 |
| **IXNW750** | 0-1 | 42 | multicore | 8/5/2015 | 20.1040 | -93.5671 | 2021 | -20.32 | 4.76 | 0.98 | 0.14 | -249.73 |
| **IXW500** | 0-1 | 41 | multicore | 8/6/2015 | 19.4441 | -93.8887 | 1010 | -20.73 | 4.69 | 1.33 | 0.17 | -116.86 |
| **IXW250** | 0-1 | 45 | multicore | 8/6/2015 | 19.4307 | -93.0950 | 583 | -21.28 | 3.75 | 1.70 | 0.20 | -144.62 |
| SL30A-100 | 0-1 | 44 | multicore | 8/6/2015 | 18.9335 | -93.3615 | 190 | -21.85 | 4.13 | 1.65 | 0.21 | -99.88 |
| SL30A-250 | 0-1 | 43 | multicore | 8/6/2015 | 19.0923 | -93.4017 | 496 | -21.39 | 4.05 | 1.68 | 0.21 | -107.55 |
| SL28-500 | 0-1 | 30 | multicore | 8/7/2015 | 19.2236 | -95.6998 | 1155 | -22.15 | 4.30 | 1.16 | 0.15 | -131.12 |

| | | | | | | | | | | | | |
|---|---|---|---|---|---|---|---|---|---|---|---|---|
| SL28-750 | 0-1 | 29 | multicore | 8/7/2015 | 19.3247 | -95.5910 | 1564 | -20.96 | 4.50 | 1.16 | 0.15 | -223.12 |
| SL30-100 | 0-1 | 35 | multicore | 8/7/2015 | 18.6970 | -94.4307 | 200 | -21.88 | 4.01 | 1.57 | 0.20 | -109.35 |
| SL30-250 | 0-1 | 39 | multicore | 8/7/2015 | 18.8575 | -94.4249 | 518 | -21.43 | 4.20 | 1.53 | 0.20 | -128.21 |
| SL30-500 | 0-1 | 37 | multicore | 8/7/2015 | 19.0644 | -94.4306 | 940 | -20.58 | 4.72 | 1.19 | 0.16 | -174.17 |
| SL26-500 | 0-1 | 22 | multicore | 8/8/2015 | 22.3803 | -97.2230 | 953 | -20.45 | 4.74 | 1.11 | 0.15 | -143.98 |
| SL27-500 | 0-1 | 28 | multicore | 8/8/2015 | 20.0827 | -96.2339 | 990 | -20.82 | 4.65 | 1.25 | 0.16 | -196.12 |
| SL27-750 | 0-1 | 27 | multicore | 8/8/2015 | 20.1212 | -96.1302 | 1522 | -20.34 | 4.95 | 1.10 | 0.15 | -217.24 |
| SL26-750 | 0-1 | 20 | multicore | 8/8/2015 | 22.4123 | -97.0886 | 1533 | -20.29 | 4.96 | 1.01 | 0.14 | -192.67 |
| **SL25-500** | 0-1 | 14 | multicore | 8/9/2015 | 24.2174 | -96.8213 | 952 | -20.47 | 4.73 | 1.14 | 0.16 | -149.86 |
| SL25-750 | 0-1 | 15 | multicore | 8/9/2015 | 24.1600 | -96.3943 | 1603 | -20.27 | 5.03 | 0.99 | 0.14 | -168.18 |
| **LT4** | 0-1 | 65 | multicore | 8/11/2016 | 18.9858 | -92.1513 | 19 | -21.31 | | | | -93.00 |
| SL29-900 | 0-1 | 32 | multicore | 8/14/2016 | 19.1524 | -94.8298 | 1648 | -21.34 | | | | -191.88 |
| SL26B-500 | 0-1 | 26 | multicore | 8/19/2016 | 20.7047 | -96.4804 | 1200 | -20.39 | | | | -337.14 |
| SL33-150 | n/a | 71 | grab | 9/19/2015 | 22.3629 | -91.6590 | 453 | -20.18 | 3.85 | 3.66 | 0.42 | -178.12 |
| EZ01 | n/a | 61 | grab | 9/21/2015 | 19.2451 | -92.2803 | 35 | -21.87 | 4.23 | 2.13 | 0.22 | -100.28 |
| EZ02 | n/a | 60 | grab | 9/21/2015 | 19.3880 | -92.2805 | 52 | -22.09 | 3.54 | 2.21 | 0.22 | -134.61 |
| EZ03 | n/a | 52 | grab | 9/21/2015 | 19.5158 | -92.5126 | 130 | -21.41 | 2.83 | 1.23 | 0.14 | -188.47 |
| SL31-100 | n/a | 51 | grab | 9/23/2015 | 19.5261 | -92.5941 | 160 | -21.34 | 3.19 | 1.55 | 0.17 | -165.27 |
| SL31-150 | n/a | 48 | grab | 9/23/2015 | 19.6081 | -92.6889 | 362 | -21.27 | 3.36 | 1.74 | 0.20 | -164.47 |
| SL31-80 | n/a | 49 | grab | 9/23/2015 | 19.4394 | -92.6793 | 160 | -21.79 | 3.00 | 1.31 | 0.14 | -270.69 |
| SL30-100 | n/a | 36 | grab | 9/24/2015 | 18.6975 | -94.4502 | 186 | -22.36 | 3.04 | 1.39 | 0.15 | -122.04 |
| SL30-150 | n/a | 38 | grab | 9/24/2015 | 18.7524 | -94.4300 | 296 | -21.53 | 3.19 | 1.29 | 0.15 | -158.96 |
| SL30-80 | n/a | 34 | grab | 9/24/2015 | 18.6662 | -94.4551 | 151 | -22.09 | 3.03 | 1.33 | 0.15 | -140.53 |
| SL26-20 | n/a | 23 | grab | 8/23/2016 | 22.3033 | -97.5660 | 51 | -21.01 | 7.06 | 0.73 | 0.10 | |
| SL26-40 | n/a | 21 | grab | 8/23/2016 | 22.3843 | -97.4629 | 77 | -20.96 | 6.20 | 1.00 | 0.13 | -202.99 |
| SL25-150 | n/a | 17 | grab | 8/24/2016 | 24.0869 | -97.1196 | 236 | -20.69 | 5.50 | 1.14 | 0.15 | -167.87 |
| SL25-20 | n/a | 18 | grab | 8/25/2016 | 24.0768 | -97.5237 | 46 | -21.07 | 7.20 | 0.88 | 0.12 | -134.24 |
| SL25-40 | n/a | 16 | grab | 8/25/2016 | 24.1004 | -97.3018 | 89 | -21.11 | 6.15 | 1.13 | 0.15 | -132.16 |
| SL25-60 | n/a | 19 | grab | 8/25/2016 | 24.0528 | -97.2236 | 126 | -21.10 | 5.36 | 1.03 | 0.13 | -155.67 |
| SL24-80 | n/a | 9 | grab | 8/26/2016 | 25.5575 | -96.3911 | 167 | -21.30 | 5.10 | 1.20 | 0.16 | -159.64 |
| SL24-100 | n/a | 10 | grab | 8/26/2016 | 25.5536 | -96.3384 | 339 | -21.11 | 4.51 | 1.26 | 0.17 | -163.60 |
| SL24-150 | n/a | 13 | grab | 8/26/2016 | 25.5455 | -96.3433 | 337 | -21.42 | 5.09 | 1.34 | 0.18 | -136.12 |

| | | | | | | | | | | | | |
|---|---|---|---|---|---|---|---|---|---|---|---|---|
| SL24-20 | n/a | 12 | grab | 8/27/2016 | 25.5455 | -96.9344 | 44 | -21.41 | 6.64 | 0.93 | 0.12 | -161.52 |
| SL24-40 | n/a | 8 | grab | 8/27/2016 | 25.5853 | -96.6187 | 78 | -21.42 | 5.33 | 0.90 | 0.12 | -180.67 |
| SL24-60 | n/a | 11 | grab | 8/27/2016 | 25.5514 | -96.4522 | 138 | -21.14 | 5.03 | 1.06 | 0.14 | -149.91 |
| SL23-80 | n/a | 4 | grab | 8/28/2016 | 26.7041 | -96.4507 | 167 | -21.25 | 4.61 | 1.10 | 0.15 | -170.15 |
| SL23-100 | n/a | 3 | grab | 8/28/2016 | 26.7490 | -96.4499 | 195 | -21.24 | 4.90 | 1.21 | 0.16 | -166.48 |
| SL23-150 | n/a | 2 | grab | 8/28/2016 | 26.7542 | -96.4114 | 271 | -21.49 | 4.91 | 1.34 | 0.18 | -142.18 |
| SL23-20 | n/a | 7 | grab | 8/29/2016 | 26.5528 | -96.9433 | 36 | -21.47 | 6.80 | 1.01 | 0.14 | -133.74 |
| SL23-40 | n/a | 6 | grab | 8/29/2016 | 26.6387 | -96.6024 | 85 | -21.24 | 4.60 | 0.82 | 0.11 | -255.96 |
| SL23-60 | n/a | 5 | grab | 8/29/2016 | 26.6578 | -96.4665 | 126 | -21.32 | 4.96 | 0.99 | 0.14 | -176.80 |
| SL22-20 | n/a | 1 | grab | 8/30/2016 | 27.4842 | -96.7632 | 45 | -21.90 | 5.70 | 0.65 | 0.09 | -200.31 |
| **94-22** | 2-3 | 64 | multicore | 2007 | 19.3128 | -92.2063 | 25 | -21.90 | | | | -110.10 |
| **89-33** | 2-3 | 53 | multicore | 2010 | 19.9994 | -92.4976 | 1330 | -20.76 | | | | -258.20 |
| **E4** | 2-3 | 40 | multicore | 8/3/2011 | 18.9402 | -93.9997 | 535 | -21.30 | | | | -138.00 |
| **E52** | 0-1 | 31 | multicore | 8/10/2011 | 19.0048 | -94.9267 | 1263 | -20.70 | | | | -200.20 |
