# Supplementary material for: The southern Gulf of Mexico: A baseline radiocarbon isoscape of surface sediments and isotopic excursions at depth
Source: PLoS One. 2020 Apr 15;15(4):e0231678. doi: 10.1371/journal.pone.0231678 (PMC7159241; doi:10.1371/journal.pone.0231678)
Supplement: S1 Fig — Cores are ordered from shallowest water depth to deepest water depth. (PDF) [file pone.0231678.s002.pdf]

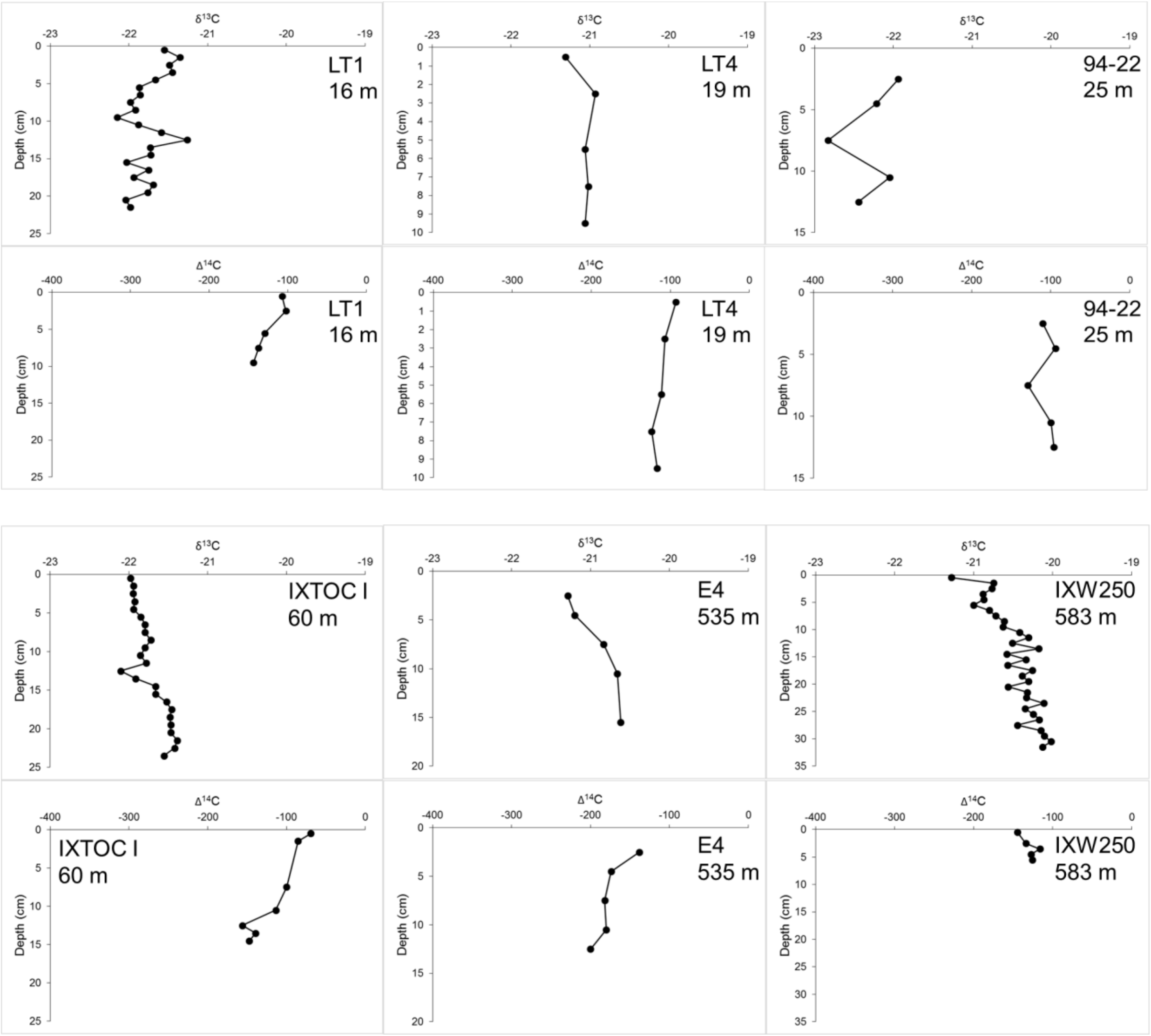

S1 Fig. Isotopic ($\delta^{13}C$ and $\Delta^{14}C$) core profiles for the remaining 18 cores examined in the southern Gulf of Mexico. Cores are ordered from shallowest water depth to deepest water depth

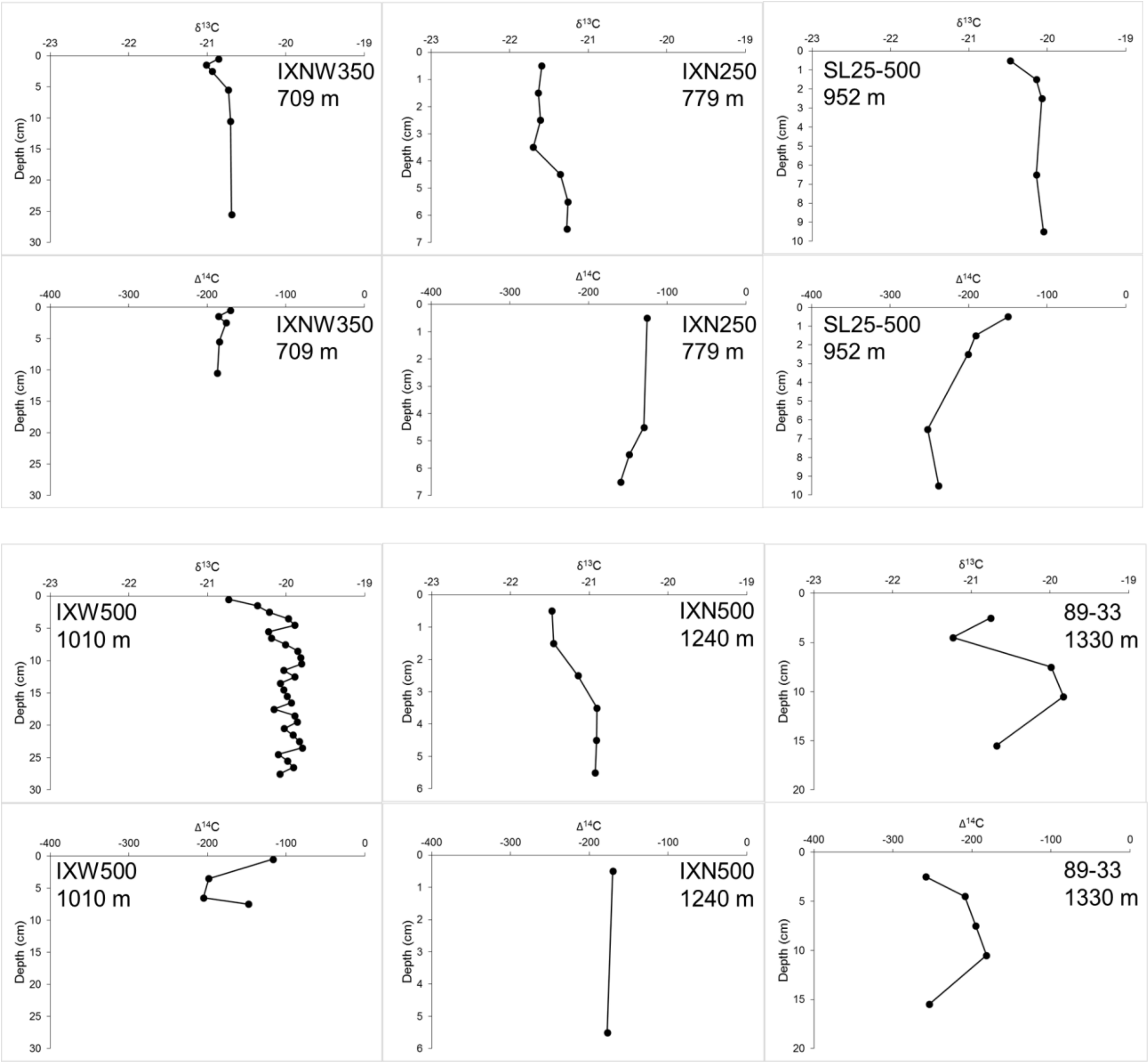

S1 Fig. Continued.

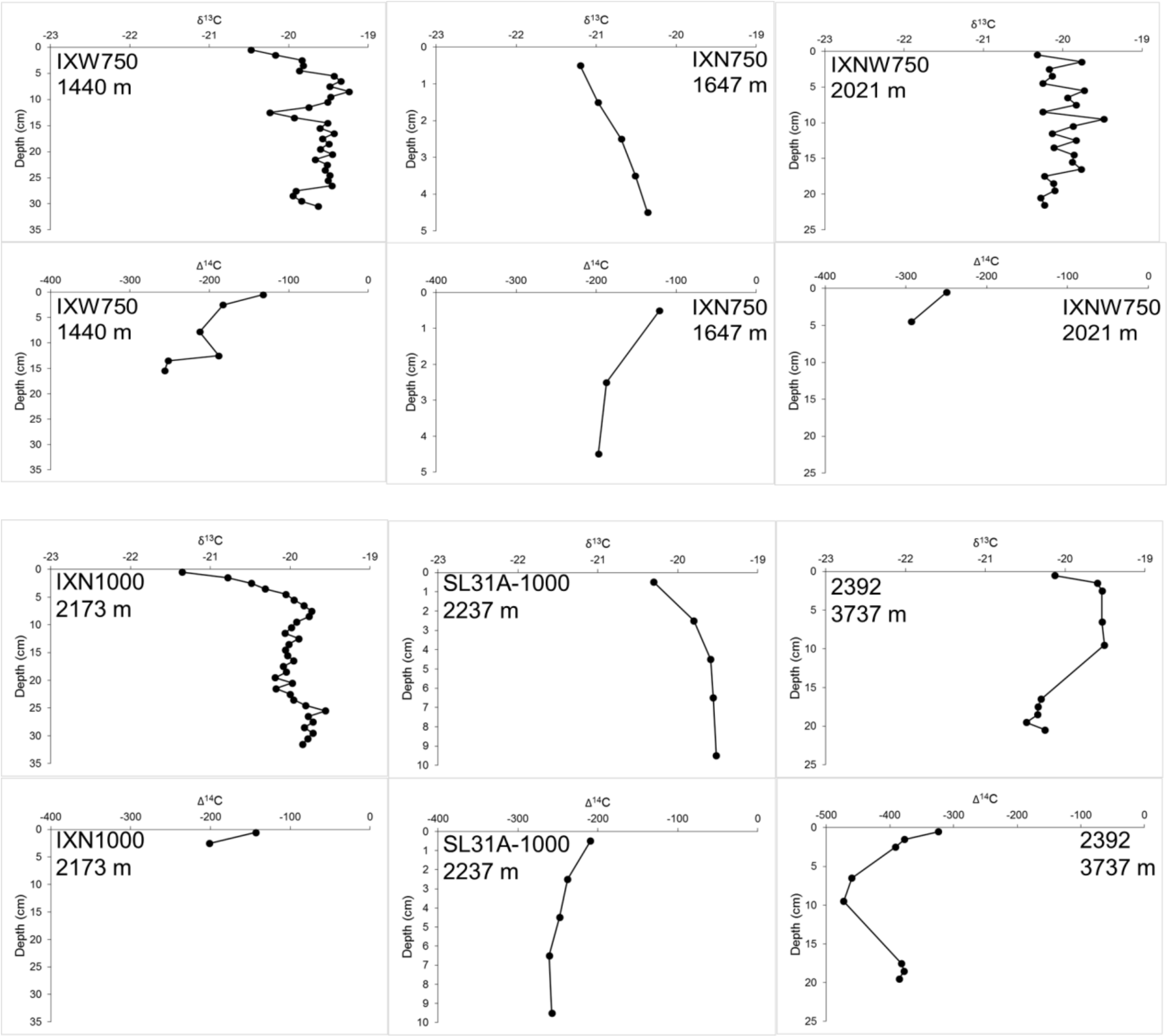

S1 Fig. Continued.
